# Cascaded Convolutional Neural Network Architecture for Speech Emotion Recognition in Noisy Conditions

**DOI:** 10.3390/s21134399

**Published:** 2021-06-27

**Authors:** Youngja Nam, Chankyu Lee

**Affiliations:** 1 Humanities Research Institute, Chung-Ang University, Seoul 06974, Korea; btoj19@cau.ac.kr; 2 Department of Korean Language and Literature, Chung-Ang University, Seoul 06974, Korea

**Keywords:** cascaded DnCNN–CNN, speech emotion recognition, residual learning

## Abstract

Convolutional neural networks (CNNs) are a state-of-the-art technique for speech emotion recognition. However, CNNs have mostly been applied to noise-free emotional speech data, and limited evidence is available for their applicability in emotional speech denoising. In this study, a cascaded denoising CNN (DnCNN)–CNN architecture is proposed to classify emotions from Korean and German speech in noisy conditions. The proposed architecture consists of two stages. In the first stage, the DnCNN exploits the concept of residual learning to perform denoising; in the second stage, the CNN performs the classification. The classification results for real datasets show that the DnCNN–CNN outperforms the baseline CNN in overall accuracy for both languages. For Korean speech, the DnCNN–CNN achieves an accuracy of 95.8%, whereas the accuracy of the CNN is marginally lower (93.6%). For German speech, the DnCNN–CNN has an overall accuracy of 59.3–76.6%, whereas the CNN has an overall accuracy of 39.4–58.1%. These results demonstrate the feasibility of applying the DnCNN with residual learning to speech denoising and the effectiveness of the CNN-based approach in speech emotion recognition. Our findings provide new insights into speech emotion recognition in adverse conditions and have implications for language-universal speech emotion recognition.

## 1. Introduction

Emotions play a crucial role in social interactions as they provide important information about the thoughts and behavior of the speaker. Speech is one of the most efficient and fundamental ways of expressing emotion [1]. The study of speech emotion recognition, which aims to identify high-level affective contents of an utterance from low-level acoustic features, has drastically evolved over the past decade with the rapid development in human–machine interactions. Speech emotion recognition has a wide applicability, including in automated call centers [2], health care [3], onboard vehicle driving systems [4], education [5], and many other smart systems [6]. However, there is a long way to go to achieve a more natural interaction between humans and machines. In particular, determining the most effective features for emotion recognition remains an open issue, which makes speech emotion recognition very challenging. In fact, emotion classification is not straightforward, even for humans.

Speech emotion recognition systems consist of two main modules: feature extraction and classification. Feature extraction is the first core processing phase, in which the most relevant features that are available in the speech signal are extracted. Prior research on feature extraction is generally classified into four categories: continuous features, qualitative features, spectral features, and Teager energy operator (TEO)-based features [7]. Continuous features include formant, timing, pitch-related features, and energy-related features [8]. Qualitative features are the perceptual correlates of the voice quality, e.g., being harsh, tense, and breathy [9]. Spectral features carrying information about the frequency contents that are present in speech are typically exploited as short-time representations of speech signals. Primary spectral features include linear predictor coefficients (LPC), linear predictor cepstral coefficients (LPCC), and mel-frequency cepstral coefficients (MFCC) [10]. TEO-based features, which are primarily employed in stress classification, include the TEO-decomposed FM variation, the normalized TEO autocorrelation envelope area, the critical-band-based TEO autocorrelation envelope area [11], and temporal feature integration for capturing the temporal dependency of successive feature observations [12].

Classification is the second core processing step in speech emotion recognition, in which the extracted features are mapped onto the relevant emotion classes. Previous studies have identified various classification algorithms to generate robust emotional features. Researchers have proposed support vector machines [13,14], Gaussian mixture models [15], hidden Markov models [16], artificial neural networks [17], the K-nearest neighbor [18], and binary decision trees [19]. These classifiers have demonstrated good emotion classification performance. However, these traditional approaches involve extracting well-established handcrafted features from signal cues, which typically requires significant human expertise and substantial effort. Furthermore, their performance varies widely across different speech databases.

Recently, deep learning has emerged as a promising research area in machine learning and has increasingly gained attention in various domains, including speech [20,21] and image recognition [22]; natural language processing [23]; and, more recently, speech emotion recognition [24,25,26]. In particular, deep neural networks (DNNs) can learn high-level invariant features of the input signal from the raw data and provide a state-of-the-art classification performance [27,28]. Jiang et al. [29] proposed a hybrid DNN architecture to address the issue of heterogeneous acoustic features that usually degrade the classification performance in speech emotion recognition. This architecture focuses on extracting informative feature representations, which are fed into a fusion deep network, and then classified using a support vector machine. Recurrent neural networks (RNNs) can remember previous inputs in internal states and learn temporal contextual information; thus, they have shown improvements in terms of classifying emotion [30].

Convolutional neural networks (CNNs) [31] utilize a succession of layers of trainable convolution filters and optional pooling operations that are applied to local features. Although they were initially designed for computer vision tasks, CNNs have been efficient in recognition tasks of one-dimensional signals, such as audio [32] and speech [33]. In [34], one-dimensional CNN (1D CNN) and two-dimensional CNN (2D CNN) architectures outperformed standard feature-based classification and temporal feature integration methods in general audio classification. CNNs have also been effectively applied in speech emotion recognition tasks. Some studies have utilized CNN-based models trained on information generated from speech signals using spectrograms to extract high-level affective features [24,35]. A unique architecture based on a merged CNN, with a 1D CNN branch and a 2D CNN branch, was proposed to learn high-level features from raw input signals and log-mel spectrograms [36]. The 1D CNN and 2D CNN are first trained; then, their learned features are repurposed and transferred to the merged CNN, which is subsequently fine-tuned. In another study [37], both spectrogram and phoneme embedding features were used as the input of a multichannel CNN model to achieve a good result on the interactive emotional dyadic motion capture (IEMOCAP) corpus. A lightweight and efficient CNN-based architecture was also proposed to learn deep frequency features for speech emotion recognition [38]. In [39], a CNN-based architecture was tested on the acted emotional speech dynamic database (AESDD) by analyzing the sequential time frames and its performance surpassed some existing techniques, which rely on handcrafted features. Very recently, Abbaschian et al. [40] comprehensively and systematically reviewed major deep learning approaches employed in the speech emotion recognition research, combined with their associated speech databases. This review indicates that CNN-based approaches play an important role in speech emotion recognition.

Therefore, CNNs have proven to be powerful for a range of speech emotion recognition tasks. In a real scenario, human speech emotion perception typically involves listening to the background noise. However, CNNs have been applied mostly on speech databases that are recorded in a controlled environment. Consequently, it remains unclear whether CNNs are effective for the recognition of emotions in noisy speech. Meanwhile, CNNs have proven to be successful in performing image denoising [41]. Very recently, a denoising CNN (DnCNN) has shown promising results for removing environmental noise and enhancing the prediction accuracy to classify the defect patterns on semiconductor wafers [42]. To the best of our knowledge, DnCNN methods have not been evaluated in the speech domain. Furthermore, research on speech emotion recognition has focused on certain languages, such as English and German [43,44]. To date, no prior studies have investigated the effectiveness of deep learning architectures, including CNN, in a Korean emotional speech database.

This study investigates the performance of a speech emotion recognition algorithm based on a DnCNN that is trained on a Korean emotional speech database. In particular, a cascaded DnCNN–CNN architecture was employed without using any traditional handcrafted features. For comparison, we investigated the performance of a baseline CNN model to further explore the capability of CNN-based architectures for speech signal denoising. The Korean database was recorded under less-controlled conditions and mixed with three types of environmental noise to simulate real-world listening situations. As the Korean database for this study contains a slight degree of noise, we also evaluated the proposed DnCNN–CNN alone on the original Korean database. This was done to further assess the effectiveness of the proposed classification method in recognizing emotions from noisy speech. In addition, we evaluated the proposed DnCNN–CNN on a popular German emotional speech database, EMO-DB. It was mixed with three types of environmental noise to further explore the feasibility of the DnCNN–CNN in emotion recognition for speech in another language. As for the Korean database, the CNN was also tested using the German speech database.

## 2. Materials and Methods

### 2.1. Database

We assessed the effectiveness of the proposed cascaded DnCNN–CNN architecture and the baseline CNN architecture using two speech emotional databases: the newly developed Chung-Ang database of Korean emotional speech (CADKES) and the Berlin Emotional Speech Database (EMO-DB) [44].

CADKES: Owing to the lack of an available Korean emotional speech dataset for this type of research, we created a dataset. Twenty-six actors (13 males and 13 females; mean age: 23.2 years; range: 19–27 years) were individually recorded in a less-controlled condition at Chung-Ang University (CAU). The actors were recorded in the presence of background noise. All actors were undergraduates of the Department of Theatre at CAU and native Korean speakers. The recordings were made using a Rode USB microphone (Model Podcaster, RODE, Sydney, Australia), which was placed approximately 20 cm from each speaker. The computer had a 16-bit audio resolution, and the software program Praat [45] was used with a sampling rate of 44.1 kHz. The recordings were later down-sampled to 16 kHz. All actors produced 52 short sentences, which consisted of 40 declarative sentences and 12 interrogative sentences. The sentences contained a full set of Korean phonemes and were each uttered with five emotional intentions: neutral, happiness, sadness, anger, and fear. This resulted in 6760 sentences (26 actors × 52 sentences × 5 emotions). The Korean sentences were presented to 10 native Korean-speaking listeners for intellectual evaluation; all were determined to be intelligible. However, some recordings contained a slight level of ambient noise.

EMO-DB: The Berlin Emotional Speech Database (EMO-DB) in German is one of the most popular databases in speech emotion recognition. This database was collected from ten professional actors (five males) who were asked to express the given text (five short and five longer sentences) with seven emotions: neutral, happiness, sadness, anger, fear, disgust, and boredom. The recording occurred in an anechoic room at the Technical University of Berlin. A human perception test with 20 German listeners was conducted to ensure the emotional quality and naturalness of the recorded utterances. The EMO-DB consisted of 535 utterances that had an emotion recognition accuracy exceeding 80% in the perception test. Within the database, the seven emotions were not equally distributed. There were 79 neutral instances, 71 instances of happiness, 62 instances of sadness, 127 instances of anger, 69 instances of fear, 46 instances of disgust, and 81 instances of boredom.

### 2.2. Noise Materials

The present study was designed to evaluate the effectiveness of the DnCNN under a real-world noise environment. To simulate noise-corrupted speech signals, we employed the Diverse Environments Multichannel Acoustic Noise Database (DEMAND) [46], which provides a set of recordings of environmental noise in a variety of indoor and outdoor settings. The DEMAND recordings were performed with a 16-channel planar array of microphones and a target length of 5 min. In this study, three types of indoor noise were selected: cafeteria noise recorded in a busy office cafeteria (PCAFETER), train station noise recorded in the main transfer area of a busy subway station (PSTATION), and subway noise recorded in a subway (TMETRO). Subsequently, the three types of noise were added to the utterances of CADKES and EMO-DB. The signal-to-noise ratio (SNR) was set to 10 dB. Each speech database was mixed with the three types of environmental noise. This mix can be expressed as z = (1 − w)x + wy, where x is the original audio signal, y is the environmental noise signal, and w is a weighting parameter.

### 2.3. Spectrogram Generation

A spectrogram is a 2D visual representation of the spectral density of an audio or speech signal (1D) as it varies with time at different frequencies. It has been demonstrated to be highly appropriate for converting a 1D speech-signal-based representation into a 2D CNN representation [47,48]. As the present study is concerned with learning high-level features directly from speech signals using a 2D CNN-based architecture without considering handcrafted features, a spectrogram was used as input data to the 2D CNN.

The spectrogram was implemented using a short-term Fourier transform (STFT). The STFT first segments a long-time speech signal into overlapping shorter frames of equal length and then is separately applied on each frame. This reveals the Fourier spectrum on each frame. The Fourier transform provides not only the frequencies present in the signal, but also the magnitude of each frequency. In brief, the spectrogram is the output of the STFT, in which the horizontal axis represents the time, and the vertical axis represents the frequency of each frame. When considering the spectrogram, the darker the color is, the higher the magnitude at a particular time–frequency point is. The spectrogram has been used extensively in speech signal analysis, including speech emotion recognition. In this study, the spectrograms were extracted with a size of 256 × 256, and they had a 50% overlap.

### 2.4. CNN

CNNs feature a multilayer feedforward architecture that typically consists of alternating convolutional and pooling layers followed by fully connected layers. The convolutional layer is a fundamental component of the CNN architecture, in which the feature extraction is performed by moving a predefined number of filters along the input. The pooling layer simplifies the output from the convolutional layer and achieves spatial invariance by down-sampling the resolution of the features. Pooling significantly reduces the computational parameters, thereby reducing the network computations. The most popular pooling algorithm is max pooling, which maintains the maximum value among the generated features and discards the others in the pooling region. The output feature maps of the pooling layer are connected to one or more fully connected layers, in which every input in one layer is connected to every output in another layer. The fully connected layer extracts the global features from the local convolutional features and performs classification on the extracted features.

### 2.5. Residual Learning

The success of CNNs has been primarily attributed to the stacking of the distinct types of layers. The general concept behind their function is that the CNN layers progressively learn more abstract and complex features. Apparently, it is reasonable to assume that, as the network goes deeper, its accuracy improves. However, empirical evidence shows that with an increasing network depth, the accuracy becomes saturated or even degrades rapidly owing to the notorious vanishing gradient problem.

The residual learning framework was initially developed to address this performance degradation problem in image recognition [41]. Within the framework, the layers are reformulated for learning residual functions with respect to the layer inputs, rather than learning the unreferenced functions. The formulation of residual learning is as follows: r=y−x, where *y* and *x* are assumed to denote the underlying desired mapping and inputs to a few stacked layers, respectively. These stacked layers are hypothesized to approximate the residual function *r*, rather than *y* [41]. Then, the original function becomes $\hat{x}=r+x$. The residual learning approach achieves a good performance for denoising tasks by effectively resolving the vanishing gradient and the degradation problem [49].

### 2.6. Denoising CNN

The concept of residual learning was subsequently adopted in the DnCNN for image denoising [50], which led to a significant increase in the advanced performance. The residual image learning strategy, r=y−x, fits the residual image *r* more quickly than the clean image x [51]. Subsequently, the modified residual learning strategy, x=y+*r*, where *r* is targeted for our noise-removal method, is finally applied to the DnCNN to directly estimate the noise present in the input image.

Most recently, Chae and Bae [42] exploited a cascade of the DnCNN with residual learning and a CNN for identifying wafer map defects. These can be caused by dust in a cleanroom and/or by problems in the fabrication process or human error. The results showed that the residual-learning-based DnCNN outperformed the baseline CNN by 10%. As an attempt to extend the cascaded DnCNN–CNN architecture to speech denoising, we can extend the concept of residual learning to emotional speech. Accordingly, we adopted the following loss function [52].

(1) $$l\left(\theta \right)=\frac{1}{2N}\overset{N}{\underset{i=1}{\sum }}||R\left(y_{i};\theta \right)-{\left(y_{i}-x_{i}\right)||}^{2}$$

where *N* is the number of noisy speech patches and R$\left(y_{i};\theta \right)$ is the residual mapping function. *θ*, $y_{i}$, and $x_{i}\;$denote the parameters of the proposed network, the noisy speech patch, and the clean speech patch, respectively.

### 2.7. Network Architecture

**Figure 1:**
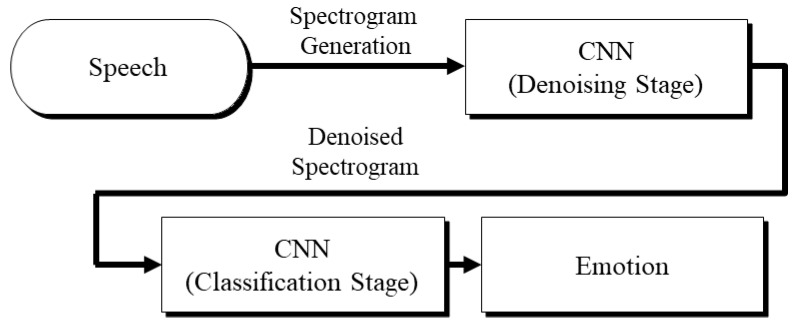
Block diagram of the proposed DnCNN–CNN architecture.

**Figure 2:**
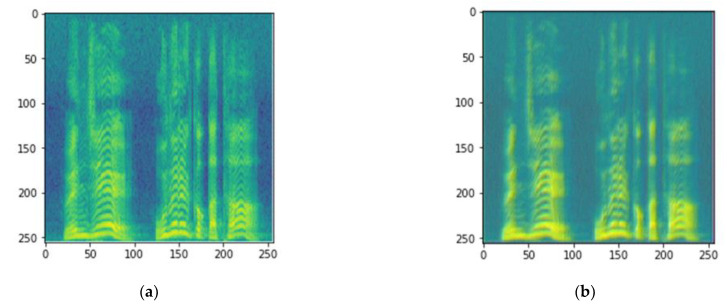
Sample spectrograms of an utterance in the Korean emotional speech database: (**a**) Original spectrogram; (**b**) Denoised spectrogram.

Figure 1 presents a block diagram of the proposed cascaded DnCNN–CNN architecture for emotional speech recognition. The cascaded architecture consists of two stages. The first stage is the denoising stage, in which the denoised spectrograms are generated by removing the noise from the noisy speech signals. Figure 2 shows the original and denoised sample spectrograms of an utterance in the Korean emotional speech database. The second stage is the classification stage, in which the CNN classifier recognizes the emotional states. Notably, this classifier serves as the baseline for comparison purposes.

This study employed a network architecture that is similar to that used in a previous investigation [42]. Table 1 provides a detailed description of our proposed network architecture. The input of the network is a 256 × 256 spectrogram that is generated from noisy emotional speech signals. The residual learning stage consists of four convolutional layers. Batch normalization layers are used after the convolutional layers to normalize the inputs of each layer [53]. During this residual learning stage, denoised spectrograms are generated by removing the noise from the noisy speech signals.

The classification stage consists of four convolutional layers, four max pooling layers, one flattened layer, one dropout layer, and two fully connected layers. All convolutional layers have 32 (3 × 3) kernels, which are applied with a stride of 1. Each convolutional layer is followed by a subsequent max pooling layer of 32 (2 × 2) kernels with a stride of 1. Then, the output of the fourth max pooling layer is flattened to a single vector of size 8192 × 1 before it is passed onto two fully connected layers with a 70% dropout. Subsequently, the output is fed into the first fully connected layer, which has 256 outputs. The second fully connected layer performs the five-class classification.

In this architecture, the rectified linear unit (ReLU) is used as the nonlinear activation function for the convolutional layers and the first fully connected layer, except for the second fully connected layer. The second fully connected layer uses the sigmoid function to squeeze the final output into the range from 0 to 1. The network parameters were trained using the back-propagation algorithm with stochastic gradient descent. We trained the network for 1000 epochs at a learning rate of 0.01. The dataset was randomly split into five subsets to perform a five-fold cross validation and the model with the best overall performance was selected for data analysis.

## 3. Results

The efficiency of the baseline CNN and the proposed cascaded DnCNN–CNN in speech emotion recognition under noisy environmental conditions was evaluated. Specifically, we evaluated the two classifiers on the Korean CADKES and German EMO-DB, which were mixed with PCAFETER, PSTATION, and TMETOR noise. As CADKES contains a slight level of noise, we further analyzed the classification performance of our proposed method on CADKES to comprehensively evaluate its efficiency. For comparison purposes, we also assessed the classification performance of the baseline CNN.

Table 2 and Table 3 present the confusion matrices for the performance of the baseline CNN and the proposed DnCNN–CNN architecture, respectively, when CADKES was mixed with the PCAFETER noise. The results show that the baseline CNN classifier achieves the highest accuracy for sadness (95.3%) and the lowest accuracy for fearful (36.1%). The CNN has accuracies of 40.0%, 42.0%, and 48.1% for anger, happiness, and neutral, respectively. Misclassification is the highest for the emotional state pair of fearful and sadness, in which fearful is misclassified as sadness 56.0% of the time. In addition, neutral (47.9%), happiness (43.0%), and anger (37.7%) are most frequently misclassified as sadness. The proposed DnCNN–CNN architecture also achieves the highest accuracy for sadness (92.7%), followed by neutral (60.5%). The DnCNN–CNN classifier also exhibits the lowest accuracy for fearful (31.4%); however, it exhibits accuracies of 47.2% and 49.2% for happiness and anger, respectively. Misclassification is the highest for the fearful–sadness pair (55.2%), in which fearful is misclassified as sadness. Neutral is also most frequently misclassified as sadness (35.3%). Happiness (34.4%) and anger (29.4%) are most frequently misclassified as sadness. The overall accuracy is 3.8% higher for the DnCNN–CNN (56.1%) than the CNN (52.3%); however, the CNN performs better for the sadness and fearful emotions.

Table 4 and Table 5 present the confusion matrices for the performance of the baseline CNN and the proposed DnCNN–CNN, respectively, when CADKES is mixed with PSTATION. The CNN classifier achieves the highest accuracy for sadness (96.3%) and the lowest accuracy for happiness and fearful (37.0%). The recognition accuracies of neutral and anger are 44.0% and 45.5%, respectively. Misclassification is the highest for the fearful–sadness pair, where fearful is misclassified as sadness 57.0% of the time. This is followed by the neutral–sadness pair (52.5%), happiness–sadness pair (45.3%), and anger–sadness pair (37.0%). The CNN achieves an overall accuracy of 51.9%. The DnCNN–CNN also achieves the highest accuracy for sadness (92.6%) and the lowest accuracy for happiness (33.9%). The accuracies of neutral, anger, and fearful are 47.9%, 44.6%, and 50.2%, respectively. The DnCNN–CNN shows parallel misclassification patterns that are also observed for the CNN; neutral (46.1%), fearful (42.4%), happiness (36.3%), and anger (30.3%) are most frequently misclassified as sadness. The DnCNN–CNN (53.9%) achieves a 2% increase in the overall accuracy in comparison with the CNN (51.9%); however, the CNN performs better for the happiness, sadness, and anger emotions.

Table 6 and Table 7 present the confusion matrices for the performance of the baseline CNN and the proposed DnCNN–CNN architecture, respectively, when the CADKES is mixed with the TMETRO noise. The results indicate that the CNN classifier exhibits the highest classification accuracy for sadness (93.8%) and the lowest accuracy for anger (47.9%). The neutral, happiness, and fearful emotions have an accuracy of 58.0%, 48.6%, and 51.7%, respectively. Fearful (42.0%), neutral (38.1%), happiness (35.7%), and anger (27.7%) are most frequently misclassified as sadness. For the DnCNN classifier, sadness is the most accurately recognized emotion (90.6%), whereas anger is the least accurately recognized emotion (50.4%). Neutral, happiness, and fearful were recognized with accuracies of 79.2%, 52.8%, and 55.8%, respectively. Fearful (35.5%), happiness (20.0%), neutral (17.2%), and anger (13.4%) are most frequently misclassified as sadness. The DnCNN-CNN (64.8%) performs better, overall, than the CNN (60.1%) by 4.7%. In addition, the proposed classifier performs better for all emotions except for the sadness emotion.

As mentioned above, we evaluated the performances of the baseline CNN and the proposed cascaded DnCNN–CNN on the original CADKES that contains a slight level of noise to examine whether the classifiers perform differently. Table 8 and Table 9 provide the emotion classification results of the CNN and the DnCNN–CNN, respectively. The results show that both classifiers have an excellent performance on the original CADKES. The CNN classifier yields an overall accuracy of 93.6%. All the emotion classes are recognized with an accuracy above 90%. The best recognized emotion is neutral (95.4%). Meanwhile, misclassification is the highest for the sadness–fearful pair, where sadness is misclassified as fearful 3.8% of the time. This is followed by the sadness–neutral pair (3.6%) and the anger–happiness pairs (3.6%). The DnCNN–CNN architecture achieves a high overall accuracy of 95.8%. Neutral, sadness, and anger (>96%) have a relatively higher classification accuracy than happiness (94.7%) and fearful (93.0%). Misclassification is the highest for the emotional state pair of happiness and anger, where happiness is misclassified as anger 3.7% of the time. These results indicate that the proposed DnCNN–CNN classifier outperforms the baseline CNN classifier in terms of the overall accuracy. In addition, the proposed classifier performs better than the baseline classifier in terms of the accuracy for every class of emotion. The highest improvement in the classification accuracy is observed for sadness (6.4%), whereas the lowest improvement in the classification accuracy is observed for happiness (0.5%).

Table 10 and Table 11 provide the confusion matrices for the performances of the baseline CNN and the proposed DnCNN–CNN architecture, respectively, when the German EMO-DB was mixed with the PCAFETER noise. The CNN exhibits the highest accuracy for boredom (100.0%), followed by sadness (72.6%) and anxiety (52.2%). Disgust, neutral, and anger are recognized as the intended emotions 37.0%, 30.4%, and 26.4% of the time, respectively. Happiness is the least accurately recognized emotion (23.9%). The highest misclassification is observed for the neutral–boredom pair, where neutral is recognized as boredom in 64.6% of the instances, followed by the happiness–boredom pair (50.7%) and the disgust–boredom pair (45.7%). The proposed DnCNN–CNN achieves the highest accuracy for boredom (92.6%), followed by neutral (83.5%). Sadness, anger, happiness, and anxiety are recognized as the intended emotions in 77.4%, 76.0%, 67.6%, and 67.2% of the instances, respectively. Disgust is the least accurately recognized emotion (65.2%). The sadness–boredom pair exhibits the highest misclassification, where neutral is recognized as boredom in 21.0% of the instances, followed by the anxiety–neutral pair, where anxiety is recognized as neutral in 17.9% of the instances. The proposed DnCNN–CNN (76.6%) outperforms the baseline CNN (48.2%) in the overall accuracy for the EMO-DB mixed with the PCAFETER noise. The DnCNN–CNN also performs better than the baseline CNN for every emotion class except for the boredom emotion. The highest improvement in the classification accuracy is observed for neutral (53.1%), whereas the lowest improvement in the classification accuracy is observed for sadness (4.8%).

Table 12 and Table 13 provide the confusion matrices for the performance of the baseline CNN and the proposed DnCNN–CNN architecture, respectively, when the German EMO-DB was mixed with the PSTATION noise. The CNN obtains the highest accuracy for boredom (100.0%) and the lowest accuracy for anger (10.1%). Sadness, anxiety, neutral, disgust, and happiness are recognized as the intended emotions in 71.0%, 38.8%, 24.1%, 23.9%, and 15.5% of the instances, respectively. The highest misclassification is observed for the neutral–boredom pair, where neutral is recognized as boredom in 70.9% of the instances. This is followed by the happiness–boredom pair (62.0%) and the disgust–boredom pair (45.7%). The DnCNN–CNN achieves the highest classification accuracy for boredom (98.8%) and the lowest classification accuracy for happiness (39.4%). Sadness (88.7%) exhibits relatively better accuracies in comparison with disgust (52.2%), anxiety (50.7%), anger (46.5%), and neutral (45.6%). The highest misclassification is observed for the neutral–boredom pair, where happiness is misclassified as boredom 53.2% of the time. Disgust (26.1%), happiness (29.6%), and sadness (11.3%) are also most frequently misclassified as boredom. Anger and anxiety are most frequently misclassified as neutral (14.0%) and sadness (23.9%), respectively. The DnCNN–CNN (59.3%) achieves a higher overall accuracy than the CNN (39.4%). More specifically, the DnCNN–CNN performs better than the CNN on every emotion class except for boredom.

Table 14 and Table 15 provide the confusion matrices for the performances of the baseline CNN and the proposed DnCNN–CNN architecture, respectively, when the German EMO-DB is mixed with the TMETRO noise. The CNN obtains the highest accuracy for boredom (98.8%), followed by sadness (87.1%), Anxiety, neutral, disgust, and anger have accuracies of 61.2%, 57.0%, 52.2%, and 38.8%, respectively. Happiness is the least accurately recognized emotion (28.2%). The highest misclassification is observed for the neutral–boredom pair, in which neutral is recognized as boredom in 39.2% of the instances. This is followed by the happiness–boredom pair (38.0%) and the disgust–boredom pair (28.3%). The DnCNN–CNN achieves the highest accuracy for boredom (93.8%), followed by sadness (88.7%). Neutral (68.4%) and anger (68.2%) exhibit relatively higher classification accuracies than anxiety (59.7%), happiness (50.7%), and disgust (50.0%). Misclassification is the highest for the neutral–boredom emotional state pair, where anxiety is misclassified as neutral 30.4% of the time. This is followed by the happiness–neutral pair, where happiness is misclassified as neutral 28.2% of the time. The proposed DnCNN–CNN (69.5%) outperforms the baseline CNN method (58.1%) in terms of the overall accuracy; more specifically, the DnCNN–CNN performs better than the CNN on every emotion class except for boredom, disgust, and anxiety.

## 4. Discussion

Recently, CNNs have achieved advanced performance for a wide spectrum of applications. In particular, CNN-based approaches are compelling in terms of recognizing emotions while analyzing recorded speech in a noise-free environment. However, very little attention has been given to their ability to recognize emotional states from noisy speech. Motivated by the effectiveness of the DnCNN for noise reduction in wafer maps and image processing, this study explored their applicability in the speech domain. This was achieved by evaluating the performance of the proposed classifier in the Korean CADKES and German EMO-DB databases under noisy environmental conditions. Specifically, this investigation proposed the DnCNN–CNN architecture, which concatenates the residual-learning-based denoising stage and classification stage. For comparison, this study also analyzed the performance of the baseline CNN. Notably, only a few studies have utilized CNNs for noisy speech emotion recognition. This study provides the first evidence for the feasibility and effectiveness of the DnCNN architecture for boosting the emotion recognition performance in speech signals. In addition, this study is the first attempt at using deep learning algorithms to classify Korean emotional speech.

The results show that the proposed DnCNN–CNN achieves a 2–28.4% increase in the overall accuracy in comparison with the baseline CNN under the PCAFETER, PSTATION, and TMETRO noise conditions. Nonetheless, the two classifiers perform poorly overall on the Korean database that was mixed with the three types of noise. When it was tested with the original CADKES, the proposed DnCNN–CNN exhibited a drastic increase in the overall accuracy. The baseline CNN also gained a high overall accuracy. These patterns indicate the effect of the noise that is contained in the original Korean CADKES in emotion classification. It also supports the effectiveness of the proposed method in recognizing emotions from speech signals that are corrupted by noise. Furthermore, the performance of the proposed method on the German EMO-DB highlights the potential efficiency of the DnCNN–CNN for recognizing emotions from noisy speech. Meanwhile, it exhibits higher accuracies than the CNN under the three noise conditions. The results of the Bayesian Wilcoxon Signed Rank test based on [54] show that the probability of higher accuracy of the proposed DnCNN-CNN than the baseline CNN is 100%.

The overall DnCNN results are conceptually consistent with those of previous research on the noise reduction of image sequences [41] and semiconductor wafer maps [42] in terms of its high potential in denoising tasks across domains. The baseline-CNN-based results are also largely consistent with those of previous studies [24,32,33,34,35,36,37,38,39]. Finally, the high accuracy of speech emotion recognition for the original Korean CADKES demonstrates the effectiveness and versatility of CNNs in a variety of recognition tasks.

However, there are some important limitations to this research. First, the performance of the proposed algorithm in emotion recognition tasks for noisy speech was tested in only two languages. It remains undetermined whether the proposed DnCNN is effective in a language-universal manner. Second, the effectiveness of the DnCNN in noisy speech emotion recognition is limited because its performance varies remarkably as a function of the noise level. In particular, the DnCNN performs drastically better on the original CADKES than the CADKES mixed with different types of noise. Further studies are required to verify the effectiveness of the proposed DnCNN–CNN on noisy speech emotion recognition tasks.

As the proposed algorithm performed considerably better when using German speech than when using Korean speech, one possible direction for future studies is to boost the denoising performance and robustness of the DnCNN–CNN on emotional speech signals to which heavy noise is added. Another future research direction is to corroborate the applicability of the proposed method by exploring whether the proposed DnCNN–CNN performs well in enhancing the recognition of noisy emotional speech in languages other than Korean and German.

## Figures and Tables

**Table 1:** Structure of the proposed cascaded DnCNN–CNN.

**Denoising Stage**			
**Layer Type**	**Output Size**	**Kernel Size**	**Stride**
Convolutional Layer 1	256 × 256 × 64	3 × 3	1 × 1
Batch Normalization			
Convolutional Layer 2	256 × 256 × 32	3 × 3	1 × 1
Batch Normalization			
Convolutional Layer 3	256 × 256 × 16	3 × 3	1 × 1
Batch Normalization			
Convolutional Layer 4	256 × 256 × 3		
Batch Normalization			
**Classification Stage**			
Convolutional Layer 1	256 × 256 × 32	3 × 3	1 × 1
Max Pooling Layer 1	128 × 128 × 32	2 × 2	1 × 1
Convolutional Layer 2	128 × 128 × 64	3 × 3	1 × 1
Max Pooling Layer 2	64 × 64 × 64	2 × 2	1 × 1
Convolutional Layer 3	64 × 64 × 128	3 × 3	1 × 1
Max Pooling Layer 3	32 × 32 × 128	2 × 2	1 × 1
Flattened Layer	131,072	-	-
Dropout Layer	70%	-	-
Fully Connected Layer 1	256	-	-
Fully Connected Layer 2	5	-	-

**Table 2:** Confusion matrix of the emotion classification results of the baseline CNN that was trained on the Korean CADKES mixed with the PCAFETER noise.

	Predicted Emotion					
True emotion		Neutral	Happiness	Sadness	Anger	Fearful
	Neutral	48.1%	0.7%	47.9%	1.7%	1.5%
	Happiness	10.4%	42.0%	43.0%	1.8%	2.8%
	Sadness	2.2%	0.7%	95.3%	0.5%	1.3%
	Anger	9.2%	6.6%	37.7%	40.0%	6.6%
	Fearful	3.2%	1.7%	56.0%	3.0%	36.1%
	Overall accuracy: 52.3%					

**Table 3:** Confusion matrix of the emotion classification results of the proposed cascaded DnCNN–CNN that was trained on the Korean CADKES mixed with the PCAFETER noise.

	Predicted Emotion					
True emotion		Neutral	Happiness	Sadness	Anger	Fearful
	Neutral	60.5%	0.8%	35.3%	2.8%	0.6%
	Happiness	14.0%	47.2%	34.4%	3.5%	0.9%
	Sadness	3.7%	0.8%	92.7%	1.9%	0.9%
	Anger	11.8%	7.0%	29.4%	49.2%	2.7%
	Fearful	6.2%	1.7%	55.2%	5.5%	31.4%
	Overall accuracy: 56.1%					

**Table 4:** Confusion matrix of the emotion classification results of the baseline CNN that was trained on the Korean CADKES mixed with the PSTATION noise.

	Predicted Emotion					
True emotion		Neutral	Happiness	Sadness	Anger	Fearful
	Neutral	44.0%	0.1%	52.5%	1.9%	1.5%
	Happiness	10.5%	37.0%	45.3%	4.3%	2.9%
	Sadness	1.3%	0.3%	96.3%	0.2%	1.9%
	Anger	8.5%	5.6%	37.0%	45.5%	3.4%
	Fearful	1.9%	0.7%	57.0%	3.5%	37.0%
	Overall accuracy: 51.9%					

**Table 5:** Confusion matrix of the emotion classification results of the proposed cascaded DnCNN–CNN that was trained on the Korean CADKES mixed with the PSTATION noise.

	Predicted Emotion					
True emotion		Neutral	Happiness	Sadness	Anger	Fearful
	Neutral	47.9%	0.5%	46.1%	1.5%	3.9%
	Happiness	17.0%	33.9%	36.3%	5.3%	7.5%
	Sadness	2.5%	0.3%	92.6%	1.1%	3.4%
	Anger	11.6%	3.4%	30.3%	44.6%	10.1%
	Fearful	3.6%	0.7%	42.4%	3.0%	50.2%
	Overall accuracy: 53.9%					

**Table 6:** Confusion matrix of the emotion classification results of the baseline CNN that was trained on the Korean CADKES mixed with the TMETRO noise.

	Predicted Emotion					
True emotion		Neutral	Happiness	Sadness	Anger	Fearful
	Neutral	58.0%	0.7%	38.1%	0.5%	2.6%
	Happiness	10.8%	48.6%	35.7%	1.8%	3.1%
	Sadness	1.8%	0.5%	93.8%	0.7%	3.1%
	Anger	10.5%	8.2%	27.7%	47.9%	5.7%
	Fearful	2.3%	2.2%	42.0%	1.8%	51.7%
	Overall accuracy: 60.1%					

**Table 7:** Confusion matrix of the emotion classification results of the proposed cascaded DnCNN–CNN that was trained on the Korean CADKES mixed with the TMETRO noise.

	Predicted Emotion					
True emotion		Neutral	Happiness	Sadness	Anger	Fearful
	Neutral	79.2%	1.4%	17.2%	0.3%	1.9%
	Happiness	20.3%	52.8%	20.0%	1.4%	5.6%
	Sadness	4.2%	0.2%	90.6%	0.7%	4.3%
	Anger	17.0%	10.7%	13.4%	50.4%	8.6%
	Fearful	5.7%	2.0%	35.5%	1.0%	55.8%
	Overall accuracy: 64.8%					

**Table 8:** Confusion matrix of the emotion classification results of the baseline CNN that was trained on the original Korean CADKES.

	Predicted Emotion					
True emotion		Neutral	Happiness	Sadness	Anger	Fearful
	Neutral	95.4%	1.4%	2.2%	0.3%	0.7%
	Happiness	2.1%	94.2%	0.8%	1.9%	1.0%
	Sadness	3.6%	1.6%	90.7%	0.4%	3.8%
	Anger	0.9%	3.6%	0.6%	93.9%	1.1%
	Fearful	2.1%	1.8%	3.2%	0.8%	92.1%
	Overall accuracy: 93.6%					

**Table 9:** Confusion matrix of the emotion classification results of the proposed cascaded DnCNN–CNN that was trained on the original Korean CADKES.

	Predicted Emotion					
True emotion		Neutral	Happiness	Sadness	Anger	Fearful
	Neutral	98.1%	0.9%	1.0%	0.0%	0.0%
	Happiness	1.0%	94.7%	0.6%	3.7%	0.0%
	Sadness	1.2%	0.6%	97.1%	0.0%	1.2%
	Anger	0.5%	1.1%	1.1%	96.2%	1.1%
	Fearful	1.9%	1.4%	1.9%	1.7%	93.0%
	Overall accuracy: 95.8%					

**Table 10:** Confusion matrix of the emotion classification results of the baseline CNN that was trained on the German EMO-DB mixed with the PCAFETER noise.

		Predicted Emotion						
True emotion		Neutral	Anger	Boredom	Disgust	Anxiety	Happiness	Sadness
	Neutral	30.4%	0.0%	64.6%	0.0%	0.0%	0.0%	5.1%
	Anger	3.1%	26.4%	24.8%	7.0%	28.7%	7.8%	2.3%
	Boredom	0.0%	0.0%	100.0%	0.0%	0.0%	0.0%	0.0%
	Disgust	2.2%	0.0%	45.7%	37.0%	6.5%	0.0%	8.7%
	Anxiety	10.4%	0.0%	19.4%	3.0%	52.2%	0.0%	14.9%
	Happiness	11.3%	2.8%	50.7%	4.2%	7.0%	23.9%	0.0%
	Sadness	1.6%	0.0%	25.8%	0.0%	0.0%	0.0%	72.6%
		Overall accuracy: 48.2%						

**Table 11:** Confusion matrix of the emotion classification results of the proposed cascaded DnCNN–CNN that was trained on the German EMO-DB mixed with the PCAFETER noise.

		Predicted Emotion						
True emotion		Neutral	Anger	Boredom	Disgust	Anxiety	Happiness	Sadness
	Neutral	83.5%	0.0%	16.5%	0.0%	0.0%	0.0%	0.0%
	Anger	4.7%	76.0%	0.8%	1.6%	3.9%	13.2%	0.0%
	Boredom	7.4%	0.0%	92.6%	0.0%	0.0%	0.0%	0.0%
	Disgust	6.5%	0.0%	15.2%	65.2%	8.7%	2.2%	2.2%
	Anxiety	17.9%	1.5%	4.5%	1.5%	67.2%	1.5%	6.0%
	Happiness	12.7%	5.6%	11.3%	1.4%	1.4%	67.6%	0.0%
	Sadness	1.6%	0.0%	21.0%	0.0%	0.0%	0.0%	77.4%
		Overall accuracy: 76.6%						

**Table 12:** Confusion matrix of the emotion classification results of the baseline CNN that was trained on the German EMO-DB mixed with the PSTATION noise.

		Predicted Emotion						
True emotion		Neutral	Anger	Boredom	Disgust	Anxiety	Happiness	Sadness
	Neutral	24.1%	0.0%	70.9%	0.0%	0.0%	0.0%	5.1%
	Anger	14.0%	10.1%	31.8%	7.0%	30.2%	3.9%	3.1%
	Boredom	0.0%	0.0%	100.0%	0.0%	0.0%	0.0%	0.0%
	Disgust	2.2%	0.0%	45.7%	23.9%	8.7%	0.0%	19.6%
	Anxiety	13.4%	0.0%	20.9%	1.5%	38.8%	0.0%	25.4%
	Happiness	9.9%	1.4%	62.0%	4.2%	7.0%	15.5%	0.0%
	Sadness	0.0%	0.0%	29.0%	0.0%	0.0%	0.0%	71.0%
		Overall accuracy: 39.4%						

**Table 13:** Confusion matrix of the emotion classification results of the proposed cascaded DnCNN–CNN that was trained on the German EMO-DB mixed with the PSTATION noise.

		Predicted Emotion						
True emotion		Neutral	Anger	Boredom	Disgust	Anxiety	Happiness	Sadness
	Neutral	45.6%	0.0%	53.2%	0.0%	0.0%	0.0%	1.3%
	Anger	14.0%	46.5%	10.9%	7.8%	8.5%	7.0%	5.4%
	Boredom	0.0%	0.0%	98.8%	0.0%	0.0%	0.0%	1.2%
	Disgust	10.9%	0.0%	26.1%	52.2%	2.2%	2.2%	6.5%
	Anxiety	11.9%	0.0%	13.4%	0.0%	50.7%	0.0%	23.9%
	Happiness	22.5%	2.8%	29.6%	4.2%	0.0%	39.4%	1.4%
	Sadness	0.0%	0.0%	11.3%	0.0%	0.0%	0.0%	88.7%
		Overall accuracy: 59.3%						

**Table 14:** Confusion matrix of the emotion classification results of the baseline CNN that was trained on the German EMO-DB mixed with the TMETRO noise.

		Predicted Emotion						
True emotion		Neutral	Anger	Boredom	Disgust	Anxiety	Happiness	Sadness
	Neutral	57.0%	0.0%	39.2%	0.0%	0.0%	0.0%	3.8%
	Anger	8.5%	38.8%	14.0%	6.2%	22.5%	8.5%	1.6%
	Boredom	0.0%	0.0%	98.8%	0.0%	0.0%	0.0%	1.2%
	Disgust	0.0%	0.0%	28.3%	52.2%	13.0%	0.0%	6.5%
	Anxiety	9.0%	0.0%	9.0%	1.5%	61.2%	0.0%	19.4%
	Happiness	16.9%	2.8%	38.0%	4.2%	9.9%	28.2%	0.0%
	Sadness	1.6%	0.0%	11.3%	0.0%	0.0%	0.0%	87.1%
		Overall accuracy: 58.1%						

**Table 15:** Confusion matrix of the emotion classification results of the proposed cascaded DnCNN–CNN that was trained on the German EMO-DB mixed with the TMETRO noise.

		Predicted Emotion						
True emotion		Neutral	Anger	Boredom	Disgust	Anxiety	Happiness	Sadness
	Neutral	68.4%	0.0%	30.4%	0.0%	0.0%	0.0%	1.3%
	Anger	7.0%	68.2%	3.9%	2.3%	6.2%	11.6%	0.8%
	Boredom	6.2%	0.0%	93.8%	0.0%	0.0%	0.0%	0.0%
	Disgust	15.2%	2.2%	13.0%	50.0%	8.7%	4.3%	6.5%
	Anxiety	23.9%	1.5%	6.0%	0.0%	59.7%	0.0%	9.0%
	Happiness	28.2%	4.2%	11.3%	1.4%	4.2%	50.7%	0.0%
	Sadness	1.6%	0.0%	9.7%	0.0%	0.0%	0.0%	88.7%
		Overall accuracy: 69.5%						

## Data Availability

The datasets generated and/or analyzed during the present study are available from the first author or the corresponding author on reasonable request.
